# Report of the Committee of Dental Chemistry

**Published:** 1880-02

**Authors:** J. L. Asay


### Report of Committee of Dental Chemistry.

BY J. L. ASAY, M. D.

Chemistry, in its entirety, is a science, in which no deviation from principles is possible; the laws which govern it are positive. We may divide it into medical, dental or any other branch as we choose, but no line of demarcation exists to distinguish one from the other, since its laws are universal and fixed.

It is therefore assumed that dental chemistry, so far as its name implies, is merely an imaginary division for special study, and relates to chemical changes, occuring in oral cavity, and the effect of inorganic substances when brought into contact with organic substances, together with the chemical nature of materials employed in dental science.

As a dissertation upon the whole branch of dental chemistry would consume much time, would be equally laborious, and lead us through interminable labyrinths, we are obliged to content ourselves, on this occasion, with certain subjects, as a precursor to its more intimate relations which may from time to time appear, ever trusting that as the discoveries of to-day are but the finger-posts that point to the triumph of the morrow, so may we be induced to patiently study the complex and varied changes occuring in the mouth that we may practice our profession intelligently and successfully.

The first of these subjects, therefore, which attracts our attention is the

### REACTION OF ORAL FLUIDS.

It will be remembered that three distinct pairs of glands, empty their secretions into the mouth, besides numerous follicular glands lodged in and beneath the buccal mucus membrane.

From experiments of Magendie, Bernard and others, it is demonstrated that the fluids of the parotid and sublingual glands are clear and watery, and contain but a small proportion of solid matter, whilst that of the submaxillary is thick and visced, and contains a larger proportion of solid matter.

With the influence of the respective salivary glands on digestion, and the important part they play upon food when taken into the mouth, to be afterwards submitted to the functions of the stomach, we have no present interest, but certain it is, that they act in different degrees of efficacy for that purpose.

The secretions from these sources are often varied in quantity, under certain influences, being augmented by the action of the masticatory muscles, the odor of savory food after a long fast, and other exciting causes; diminished by fear and exalted temperature of the body.

What is considered the normal characteristics of saliva, is a question, to many minds, of perplexity, yet it has in a measure, been conceded to depend upon the nationality, habits and pursuits of the individual, and that saliva is normal, in which acidity and alkalinity in the mouth are so neutralized by contact with each other in such proportions as to leave alkalinity in excess.

A sweeping assertion has been made, and often reiterated, that the characteristic reaction of saliva is alkaline, to this we agree, so far as an admixture of the fluids is concerned, yet is far from truth as regards the identity of each distinct secretion, besides many modifications may and frequently do occur, for instance, saliva changes its chemical proportions, after a full meal, when it is alkaline, to the end of a long fast when having passed from the alkaline condition to neutrality, becomes decidedly acid in its reaction, and how shall we determine that the one condition for the time-being is not equally as normal as the other.

Again, we find from observation that in subjects of robust health and hearty eaters of wholesome food the mixed fluids of the mouth are always alkaline, but in those of effeminate stature, with nervous temperaments and sedentary habits, they are either neutral or acid.

We therefore assume that an alkaline reaction is not universal in health, even when the litmus is placed directly under the tongue, which is the most favorable position for the test. But may not the secretions be acid in one part of the mouth and alkaline in the other? Experiments have shown that where the test has been applied under the tongue in an admixture of saliva, showing an alkaline result, yet by applying the litmus to the free margin of the gums, or in a deep sulcus of the mouth, previously rendered perfectly devoid, of food or other extraneous matter, we secure a decided acid reaction.

So far as our observations have been made, we are led to believe that this acid condition is produced by the agency of the buccal mucus containing a peculiar principal called ptyalin albuminous in character, and in such condition as to be capable of producing fermentation. Nither pair of salivary glands, according to the researches of Cl. Bernard, can by themselves effect the change, "but an admixture is necessary for the generation of this peculiar ferment."

Saliva is liable to a departure from its normal standard through such a cause, as in fever, or other acute affections of the system where the degree of temperature of all the organs is raised, thus affording increased facilities by the influence of heat and moisture for the process of fermentation, and it is in such cases that an alkaline wash for the mouth is particularly grateful to the patient, and serves as a preventive to those morbid changes which attack the teeth and appendages during such a condition of exaltation.

Acids are generated in the oral cavity from food and condiments taken into it. More especially is this the case in uncleanly mouths. In such instances as where portions of the food are allowed to be retained about the teeth and in their interstices, we have by the simple process of fermentation the generation of acetic acid, and as decomposition advances, it will frequently be observed that upon removing extraneous matter from beneath the teeth or other receptacle, the familiar odor of sulphretted hydrogen is present, and such conditions will remain so long as the exciting cause is permitted to exist.

Other acids found in the mouth are lactic, citric malic, and frequently the mineral acids. The presence of these vegetable acids is attributed to their being set free during the mastication of the fruits containing them. Lactic acid exists in the mouths of those afflicted with gout, rheumatism, malarial affections and gastric complications. Mineral acids, though no doubt frequently generated in the mouth, yet chiefly owe their presence to their administration as medicines, and when we remember that the nitro-muriatic is a tonic of par-excellence in many cases, and is habitually used as such, and that hydrochloric, in the form of tinct. chloride of iron, is the sheet anchor in diptheritic affections, we need not be surprised at the ravages of tooth structure as the result of these acids.

But as I have before stated, they may be generated in the mouth. For instance, nitric acid may be formed from the nitrogenous food remaining in the mouth and concealed in recesses, the food giving up its nitrogen, which, uniting with hydrogen in the necessary equivalents to form ammonia, which, in its turn, becoming decomposed, and an oxide of nitrogen formed, of course nitric acid will be the result.

In organic structures, sulphur is always united with albumen, and the same causes that produce sulphuretted hydrogen in the mouth will assist in forming sulphuric acid.

Hydrochloric acid is contained in the gastric fluids, but the quantity that would be found in the mouth from this source as by eructation, is so trifling that its consideration will not at present be attempted; besides the salivary glands, either by their secretory or excretory functions frequently furnish it, but its presence is oftener due to the union of hydrogen with chlorine, the latter being liberated from the chlorides dissolved in the mouth or from the continued use of saline diet. Again, its presence may be due in consequence of galvanic action caused by the insertion of different metals in teeth of the same mouth. More particularly should such fillings be of gold and others of amalgam. Mucus and Saliva contain the chlorides of sodium and potassium, and these being resolved into their elementary principles by galvanic action thus established, the chlorine unites with the hydrogen, and hydrochloric acid is the consequence.

In regard to formation of acids by fermentation and habits of negligence, the old adage that "cleanliness is next to godliness," is in such cases particularly applicable, and should be deeply impressed upon the minds of those entrusting themselves to our care. The mouth needs washing as much as the face and other parts of the body. Teeth of good structure, if kept clean, seldom have their continuity impaired, otherwise we always have disintegration of tooth structure, the deprivation of its earthy constituents, and eventually of its irreparable loss.

All acids found in the mouth when predominating, although in their weakest condition, will dissolve the lime salts, and this degree of decalcification is in proportion to the density of structure attacked, and is exhibited in the greatest measure where chalkiness of the tooth is in excess from such causes as inheritance or impoverished condition of vital forces.

Acids to disintegrate tooth structure must be constant in their application. When acid first attacks a base, a salt is formed, which is no longer acid nor alkaline, but neutral in its character, and this salt must be removed, as it necessarily is, by being taken up and held in solution by the fluids of the mouth, and a new quantity of acid brought into contact with the exposed structure to continue the process of decalcification. In other words, there must be a continuous circulation of fresh acid, otherwise the process of chemical destruction is retarded and at length entirely checked. Therefore, decalcification is in exact ratio to the amount of chemical force applied.—*Trans. Cal. State Dental Asso.*

